# High-Resolution Genome-Wide Mapping of Chromatin Accessibility and Torsional Stress

**DOI:** 10.1101/2024.10.11.617876

**Published:** 2024-10-13

**Authors:** Porter M. Hall, Lauren A. Mayse, Lu Bai, Marcus B. Smolka, B. Franklin Pugh, Michelle D. Wang

**Affiliations:** 1 Department of Physics & LASSP, Cornell University, Ithaca, NY 14853, USA; 2 Howard Hughes Medical Institute, Cornell University, Ithaca, NY 14853, USA; 3 Department of Biochemistry and Molecular Biology, Pennsylvania State University, University Park, PA 16802, USA; 4 Department of Physics, Pennsylvania State University, University Park, PA 16802, USA; 5 Department of Molecular Biology and Genetics, Cornell University, Ithaca, NY 14853, USA; 6 Weill Institute for Cell and Molecular Biology, Cornell University, Ithaca, NY 14853, USA

## Abstract

Torsional stress in chromatin plays a fundamental role in cellular functions, influencing key processes such as transcription, replication, and chromatin organization. Transcription and other processes may generate and be regulated by torsional stress. In the genome, the interplay of these processes creates complicated patterns of both positive (+) and negative (−) torsion. However, a challenge in generating an accurate torsion map is determining the zero-torsion baseline signal, which is conflated with chromatin accessibility. Here, we introduce a high-resolution method based on the intercalator trimethylpsoralen (TMP) to address this challenge. We describe a method to establish the zero-torsion baseline while preserving the chromatin state of the genome. This approach enables both high-resolution mapping of accessibility and torsional stress in chromatin in the cell. Our analysis reveals transcription-generated torsional domains consistent with the twin-supercoiled-domain model of transcription and suggests a role for torsional stress in recruiting topoisomerases and in regulating 3D genome architecture via cohesin. This new method provides a potential path forward for using TMP to measure torsional stress in the genome without the confounding contribution of accessibility in chromatin.

The double-stranded helical structure of DNA inherently results in the generation of torsional stress during a host of basic processes, including replication and transcription. Many essential motor proteins, such as RNA polymerase (RNAP), track the helical groove during translocation, producing a relative rotation between the motor and the DNA. If RNAP rotation is restricted during transcription, DNA itself must twist, unwinding DNA to generate (−) supercoiling behind the polymerase, while overwinding DNA to generate (+) supercoiling in front, as described by the twin-supercoiled domain model of transcription^1–6^. Excessive torsional stress generated during transcription may, in turn, present challenges to motor progression^2,3,5–7^ and topoisomerases, a class of enzymes that can relax torsional stress and alter DNA topology, are essential to ensure cell cycle progression and are required for efficient transcription to occur^8–12^. However, residual supercoiling may offer advantages to cell survival. For example, negative torsional stress may promote processes that require DNA unwinding, such as transcription initiation, replication, or DNA repair^6,13–15^. Positive torsional stress, on the other hand, may dissociate proteins ahead of advancing motors^16^ and promote genome organization^17–19^. Overall, this suggests that genomic transactions and torsional stress generation may regulate each other. The complexity of their interplay highlights the need for sensitive techniques to investigate torsional stress across the genome *in vivo*.

Elucidating the impact of torsional stress in cells requires accurate mapping of torsion. This requires a sensor that ideally should interact with the DNA to report its torsional stress, have fast and tunable binding kinetics for capturing a snapshot of the cellular state, be readily delivered into the cell, and have a robust readout. These requirements are nearly all met by 4,5ʹ,8-trimethylpsoralen (TMP), a small photoreactive DNA intercalator that can cross-link to DNA upon UVA irradiation, with the extent of cross-linking modulated by TMP concentration and UVA dosage^20^. Because TMP unwinds DNA upon intercalation, its binding affinity, and thus the probability of generating interstrand crosslinks (ICLs) at a given site, is influenced by local torsional stress. Previous studies suggest that the relationship between torsion and the crosslinking rate is approximately linear across a relevant range of torsional stress ^21,22^. While psoralen shows great promise for mapping torsional stress, significant challenges remain to be addressed before its full utilization can be realized.

Previous studies have laid the foundation for using TMP and other psoralen derivatives as torsional sensors^21,23–26^. However, factors besides the torsional state influence the resulting photo-binding distribution^21^. Importantly, the torsion signal mapped using TMP contains a zero-torsion baseline that varies across the genome, and this baseline is dependent on DNA sequence and TMP accessibility. Establishing this baseline is crucial for differentiating between (−) torsion and (+) torsion states across the genome, and in the absence of the zero-torsion baseline, the contribution from torsion to TMP intercalation may be conflated with TMP accessibility^27^. However, it is challenging to establish such a baseline as experiments must be performed under conditions that preserve both the chromatin state and the binding states of other proteins. Thus, interpreting the psoralen sensor readout requires a method to establish the zero-torsion distribution, and such methods have yet to be firmly established.

The impact of underlying DNA sequence on the zero-torsion psoralen binding landscape has been previously addressed by comparing the results from psoralen photo-binding in the cell to that of purified genomic DNA^21,23,24,26,27^. While this method corrects for the intercalation sequence-dependence, it does not account for the reduction of photo-binding due to restricted accessibility caused by bound proteins or chromatin. Indeed, previous studies have shown that chromatin reduces global psoralen crosslinking, suggesting that local effects of accessibility may be even more pronounced^21^. One method to assess the zero-torsion baseline is to digest the DNA using a DNA-nicking enzyme^25^. Still, this approach cannot ensure the preservation of the DNA or chromatin state before psoralen photo-binding. Other methods modulate RNAP or topoisomerase activities to generate a comparative map of torsional stress to extrapolate what might be a zero-torsion state but do not solve the problem at its core^23–25^. Importantly, alteration of normal cellular functioning can, in turn, alter DNA accessibility or the zero-torsion state. These complications give a clear motive for devising a strategy of measuring the zero-torsion baseline of TMP to reveal a more accurate signal of torsional stress.

To directly address this limitation, we report a generalizable method that enables psoralen mapping on relaxed DNA *in situ* while preserving the chromatin state at high resolution. We found that the zero-torsion baseline contains significant contributions from individual nucleosomes within genes. After removing this baseline, we obtained a torsion signal that identifies regions of the genome under (−) torsion or (+) torsion. This revealed the twin-supercoiled domain model of transcription and allowed examination of how torsion may facilitate the recruitment of topoisomerases. This method provides a potential path forward for measuring torsional stress using psoralen-based methods in any cellular context.

## A method to establish the zero-torsion baseline

**Figure 1.**
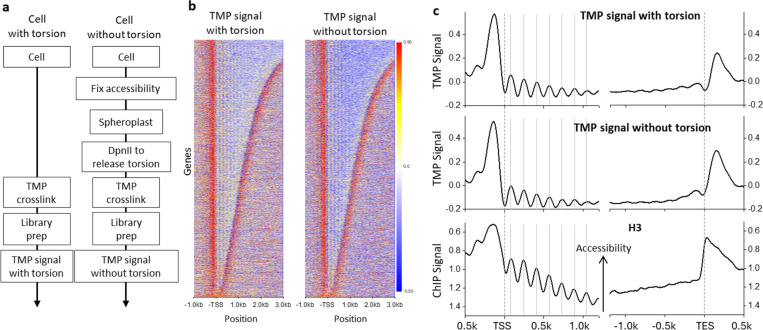
Measurement of the zero-torsion TMP baseline. (a) Strategy for determination of the TMP signal with and without torsion. (left) Cells with torsion are crosslinked with TMP prior to library preparation and then TMP signal measurement. (right) To measure the TMP baseline signal without torsion, the native-bound protein landscape is fixed by formaldehyde, and the torsion is released via DpnII digestion of the spheroplast cells before TMP crosslinking. TMP crosslinking of both samples are then mapped using Illumina-based sequencing to generate the TMP signal (methods). (b) Heatmaps of TMP signals relative to the TSSs of 5,925 genes for both the TMP signal with torsion (left, *n* = 2 biological replicates) and the TMP signal without torsion (right, *n* = 3 biological replicates) with rows sorted by the ORF size. The TMP signal from purified DNA was removed from each signal. (c) (top) Average TMP signals with and without torsion from data in (b) relative to the TSS (left) and relative to the TES (right). For comparison, previous H3-ChIP-MNase-Seq data are also shown (bottom). Only signals within the ORF of each gene were considered during averaging beyond the TSS or behind the TES.

To establish the zero-torsion baseline for the psoralen signal, we must establish a method to account for psoralen’s sequence preference and accessibility without torsion under conditions that preserve the native-bound protein landscape. To do this, we developed a method to prepare the chromatin zero-torsion state *in situ* and applied psoralen photo-binding. Formaldehyde preserved the chromatin landscape and transiently bound proteins (Methods) while stopping any subsequent cellular processes. The fixed cells were permeabilized so enzymes could diffuse into the nucleus. For *S. cerevisiae*, the cell wall was gently digested to create spheroplasts. To release torsion in intact spheroplast nuclei, we utilized DpnII, chosen for its high cut site density but inability to processively digest DNA, thus maintaining psoralen’s crosslinking ability. Upon DNA digestion, we observed an average fragment size of ~2 kb, sufficiently short to release torsion but long enough to provide a chromatin substrate for psoralen binding. This zero-torsion state preparation was subsequently crosslinked using 365-nm light, enabling measurement of the torsion-free TMP intercalation landscape (referred to as TMP signal without torsion). The *in vivo* measurement (TMP signal with torsion) was performed by directly adding TMP to the yeast media since unmodified TMP is cell-wall permeable. Cell permeability enables photo-crosslinking to be performed before cells are harvested, thus capturing native cell activity (Fig. 1a).

To locate psoralen crosslinks on purified DNA, we utilized an exonuclease-based enrichment strategy followed by an Illumina-compatible library prep, similar to TMP-seq^21,24^. Exonuclease-based enrichment relies on interstrand crosslink (ICL) formation, which prevents DNA denaturation before digestion by the single-strand-specific ExoI. We use the digested fraction to estimate the crosslinking density and provide quality control for ICL enrichment over the background. As controls, we found that libraries prepared without psoralen photo-binding showed a very low signal level (Fig. S1). By tuning the UV dosage, we ensured that the average densities of the *in vivo* and zero-torsion measurements were comparable at ~0.1–0.25 ICLs/kb (Fig. S1). If ICL density is too low, the signal-to-noise ratio is poor; if ICL density is too high, there can be library preparation artifacts (Fig. S2). ICL densities within the range used display remarkably similar signals (Fig. S1). This allows the chromatin zero-torsion baseline and the *in vivo* state to be compared and obtain an accurate genomic map of psoralen binding due to the average torsional stress. In addition, we conducted a control experiment using purified DNA to assess the extent of psoralen crosslinking due to DNA sequence preference. We can then remove this contribution during an intermediate analysis step to isolate the psoralen intercalation preference with and without torsion.

## TMP allows the mapping of positioned nucleosomes

To evaluate the contributions of DNA accessibility to the TMP intercalation profile, we examined the nucleosome positions near promoters, which are known to be well-positioned^28,29^. We expect TMP to preferentially intercalate in linker DNA regions between nucleosomes in both the *in vivo* and zero-torsion samples (Fig. S3). The ability to reveal these well-positioned nucleosomes provides a measure of the quality of our TMP data. Thus, we examined the data with and without torsion at the transcription start sites (TSSs) and observed a striking similarity between these measurements (Fig. 1b). This demonstrated that the *in vivo* psoralen signal is likely dominated by accessibility. Thus, TMP intercalation preference due to torsion may represent a smaller contribution to the overall signal than protein-mediated DNA accessibility. Therefore, using the TMP intercalation signal alone to indicate torsion may be prone to interpretation artifacts.

For both datasets, we detected a peak upstream of the TSSs, with the peak amplitude being slightly larger for the *in vivo* condition. This peak coincides with the nucleosome-free regions (NFRs), which should be more accessible to TMP (Fig. 1c). Within the gene bodies, we also observed a clear ~160 bp periodic signal, similar to that of the positioned nucleosomes near the promoters (Fig. 1c). However, the periodic pattern anti-correlates with H3-ChIP seq data, indicating that psoralen prefers to bind between nucleosomes or in NFRs (Fig. 1c)^30^. The decay in the signal amplitude away from the TSS mirrors the decay in the signal amplitude of H3-ChIP-seq, reflecting the increased variability in nucleosome positioning further from the promoter^29^. Immediately downstream of the transcription end sites (TESs), we observed a smaller peak than that before the TSSs. This smaller peak is consistent with the presence of the 3’ NFR, where transcription termination and nucleosome remodeling may occur^29,30^.

These findings show the TMP intercalation distribution reflects many features previously observed using other chromatin accessibility assays. When compared to established methods, including MNase-seq, DNase-seq and ATAC-seq, the TMP method has comparable or higher resolution^31–33^. This is likely due to the small size of TMP, which allows the detection of regions between bound proteins. Thus, the TMP method can be an excellent alternative for mapping the chromatin landscape at high resolution.

## Twin-supercoiled domains of transcription

**Figure 2.**
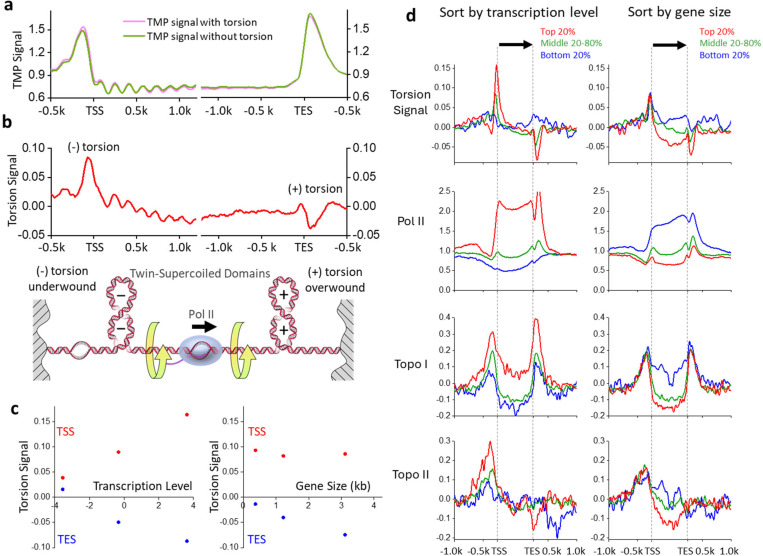
The twin-supercoiled domains of transcription. (a) Average TMP signals with and without torsion using data similar to those of Fig. 1c, replotted for easy comparison. (b) The torsion signal, defined as the difference between the TMP signal with torsion and the TMP signal without torsion. The bottom cartoon shows the twin-supercoiled domain model of transcription. (c) Average maximum and minimum torsion signals near the TSS (red) and TES (blue) as functions of the average gene expression level (left) or gene size (right) within the percentile groups specified in (d). (d) The torsion signal of genes sorted by transcription level and gene size, with genes grouped by percentiles of mRNA abundance and ORF size, respectively. Positions within the gene body are scaled by the gene length while preserving the average signal amplitude (Methods). The torsion signal is compared to previous data of Pol II (Rpb3-ChIP-Exo), topo I (Top1-ChIP-Exo), and topo II (Top2-ChIP Exo).

To determine psoralen intercalation from DNA torsion, we subtracted the zero-torsion baseline from the *in vivo* distribution, eliminating contributions from sequence preference and accessibility (Fig. 2a,b). The resulting signal has a ~5 fold reduced amplitude and should reflect contributions solely due to torsional stress. Most of the nucleosome signal was abolished within the gene bodies, indicating that most accessibility bias has been removed, although a small residual remained (Fig. 2b). We refer to the corrected signal as the “torsion signal,” with a positive value indicating (−) torsion, which facilitates psoralen intercalation, and a negative value indicating (+) torsion, which restricts psoralen intercalation. Using this approach, we examined whether we could detect the torsional state more accurately during transcription. To examine the torsion distribution along a gene, we focused on regions near promoters and terminators. We found the torsion signal now reveals (−) torsion upstream of promoters and (+) torsion downstream of terminators (Fig. 2b). This may be interpreted as a statistical average of Pol II molecules, which have elongated to different locations along the gene, each generating (−) torsion behind and (+) torsion ahead, according to the twin-supercoiled-domain model (Fig. 2b).

Thus, the torsion signals from different Pol II molecules cancel within the gene, except at the two edges of a gene region. We also found that torsion is confined to within 1–2 kbp of the promoters and terminators, supporting previous findings that transcription-dependent dynamic supercoiling is a short-range genomic force^23^.

To further examine whether the torsion signal is due to transcription, we classified genes into percentile groups based on their expression level (Fig. 2c,d). We expected that the average transcription-generated torsional stress should increase with increased gene expression. Thus, we sorted the genes based on their expression level according to previous mRNA-seq data^34^. Consistent with this prediction, genes in the bottom 20% of the expression level do not display a significant peak near the promoters nor a significant valley near the terminators. As the gene expression level increases, the torsion signal amplitudes near the promotors and terminators monotonically increase on average (Fig. 2c,d). As expected, gene expression strongly correlated with Pol II presence along the genes (based on previous ChIP-seq data of Pol II^30^). This shows that active transcription accumulates torsional stress near highly transcribed genes, supporting earlier findings from *in vitro* and *in vivo* studies^2,4–7,35–37^.

The persistence of torsion near the genes, even with full native topoisomerases, indicates that topoisomerases cannot fully keep up with transcription activities. To examine the correlation of torsional stress with topoisomerase activity, we analyzed previous ChIP-seq data of topoisomerase I (topo I) and topoisomerase II (topo II)^30^, which are the primary enzymes for relaxing transcription-generated torsion. Topo I binds near promoters and terminators, and its enrichment level correlates with an increase in the torsion signal (Fig. 2d). In contrast, topo II binds near promoters but not near terminators. As the torsion signal increases, the topo I and topo II enrichment levels also increase, suggesting that torsion might facilitate topoisomerase recruitment or retention. Unlike topo I, which binds to one DNA segment, topo II relaxes DNA via a strand-passage mechanism^38^ and has been found to prefer binding to a DNA crossover^39–42^. Because the DNA in a nucleosome wraps around the histone octamer in a left-handed fashion, the nucleosome conformation exhibits a chiral response to DNA supercoiling^43,44^. While (−) torsion readily enables DNA crossing formation via the juxtaposition of the entry and exit DNA segments in a nucleosome, (+) torsion displaces entry and exit DNA segments before bringing them together again, buffering torsional stress^43^. Thus, there is less DNA crossing at a nucleosome available to allow topo II binding. Consistent with this interpretation, we previously found that topo II has a faster relaxation rate and is more processive on chromatin under (−) supercoiling than under (+) supercoiling^39^. Thus, the absence of topo II near the terminators may not be due to any active exclusion of top II at those regions but is a natural consequence of the interplay between the DNA chirality and nucleosome chirality.

Previous studies suggest torsion accumulates more significantly over longer genes^5^. As Pol II elongates, its rotation may be more restricted with a lengthening in the RNA transcript, which is known to be associated with large machinery, such as spliceosomes^1,45–47^. Thus, torsional stress will significantly increase toward transcription termination for longer genes. We grouped data based on gene size (Fig. 2c,d), and consistent with this prediction, the (−) torsion near the promoters remained unchanged with an increase in gene size, but the (+) torsion near the terminators increased with gene size to a moderate extent. As expected, we found no significant dependence of topo I or topo II enrichment on gene size near the promoters. However, we noticed a similar lack of dependence near the terminators, likely due to the moderate dependence of torsion on gene size.

## Torsion accumulation between genes

**Figure 3.**
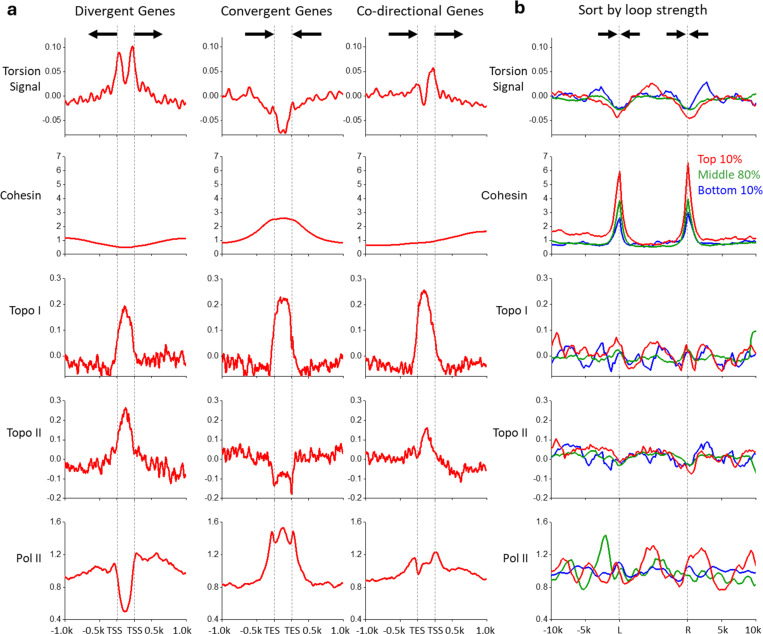
Torsion between genes and the maintenance of 3D genome structure. (a) The torsion signal plotted for three gene-pair configurations: divergent (left, 1275 pairs), convergent (middle, 1364 pairs), co-directional (right, 2426 pairs). The torsion signal is compared to previous protein mapping data of the same gene-pair configurations for cohesion (SccI-Chip), topo I (Top1-ChIP-Exo), and topo II (Top2-ChIP Exo), and Pol II (Rpb3-ChIP-Exo). (b) The same signals as in (a) plotted across 527 previously detected mitotic loops, grouped by percentiles of the loop score. Positions between the two loop boundaries (L and R) are scaled by the DNA length in between while preserving the average signal amplitude (Methods).

Transcription-generated torsion may dissipate over distance due to topoisomerase relaxation and DNA end rotation^6,23,48^. If so, torsion generated from one gene will most likely impact the torsional state of neighboring genes. For example, (−) torsion may more likely accumulate near promoters of two divergent genes, while (+) torsion may more likely accumulate near terminators of two convergent genes. To examine this prediction, we plotted the torsion signal in three gene-pair configurations (Fig. 3a). By rescaling the intergenic region between nearest neighbors, we mapped the average behavior of torsional stress in these regions. We found that between promoters of two divergent genes, the torsion signal rises throughout the intergenic region, consistent with (−) torsion accumulation between these gene pairs. Conversely, we observed that between terminators of converging genes, the torsion signal drops throughout the intergenic region, consistent with (+) torsion accumulation between these gene pairs. When two neighboring genes are oriented codirectionally, the torsion signal drops after the terminator of the trailing gene and rises before the promoter of the leading gene. However, the amplitude of the torsion signal is smaller in the codirectional-gene configuration than in either the convergent-gene or divergent-gene configurations, indicating that (+) torsion and (−) torsion may cancel in those regions. By grouping neighboring genes in these configurations, we demonstrate how torsion accumulates between neighboring gene pairs.

Since transcription-generated torsional stress hinders Pol II progression, topoisomerases must be recruited to active genes to resolve the torsional stress. To examine where Pol II, topo II, and topo II are located, we analyzed previous ChIP-seq data of Pol II, topo I, and topo II^30^. We show that Pol II has a minimal presence in the intergenic region between divergent genes and is enriched in the intergenic region between convergent genes, consistent with a previous finding that after termination, Pol II tends to remain on the DNA downstream of the terminator^30,34^. We also find that topo I is enriched in all intergenic regions with significant (+) torsion or (−) torsion, indicating that topo I effectively targets regions under torsional stress. In contrast, topo II localizes to the intergenic region between divergent genes but is depleted from the intergenic region between convergent genes, indicating that topo II prefers regions with (−) supercoiling but is restricted from regions with (+) supercoiling, further supporting our interpretations. These data further demonstrate how topo I and topo II may differentially target diverse environments in the genome.

## The role of torsion in genome structure

Maintenance of three-dimensional genome structure is a crucial step in cellular function. Recent yeast genome studies have found mitotic loops near the boundaries of convergently oriented gene pairs^17,18,49^. This suggests that (+) torsional stress may regulate genome folding. We examined the signal near loop boundaries (Fig. 3b). by aligning all loop boundaries and grouped signals based on the looping strength of previously published MicroC-XL Data.^49^ This sorting method showed that cohesin^18^ is enriched at the two-loop boundaries as expected. Using this sorting method, we found (+) torsion signal at the loop boundaries, which also coincided with regions of convergent gene pairs. We found that (+) torsion increased correlated with strengthening cohesin loop boundaries, suggesting that (+) torsion in these regions may facilitate cohesin recruitment. Interestingly, in contrast to the strong topo I enrichment between all convergent gene pairs (Fig. 3a), there is no detectable topo I enrichment at the loop boundaries, constituting a subset of all convergent gene pairs. It is possible that loop formation by cohesin induces significant chromosome condensation that may exclude topo I binding. In addition, although topo II is depleted between all convergent gene pairs (Fig. 3a), this depletion is reduced at the loop boundaries, which may reflect the need for topo II in these regions since previous studies show that topo II is essential for chromosome condensation during mitosis^50,51^. These data provide evidence that the (+) supercoiling generated by transcription may facilitate genome folding in coordination with other participating proteins.

## Discussion

This work developed a method to profile the *in situ* zero-torsion baseline of psoralen-based mapping of torsional stress in yeast. The approach enabled high-resolution mapping of the psoralen intercalation distribution on torsionally relaxed DNA, while preserving the protein-bound state of chromatin. This preservation is crucial to correcting the psoralen-based torsion map because it allows the removal of the contributions of both chromatin accessibility and DNA sequence.. Through this process, we found that the psoralen technique is excellent at generating high-resolution accessibility maps of chromatin, resolving positioned nucleosomes at a resolution superior to that of ATAC seq.^32^ This demonstrates the potential utility of psoralen well beyond torsional measurements.

Our resulting torsion signal also reveals a characteristic torsion distribution of the twin-supercoiled-domain model of transcription with (−) torsion behind the promoters and (+) torsion in front of the terminators. We demonstrated how torsion increases with gene expression, and topoisomerases are recruited to regions with torsion. We also provided an explanation for the differential enrichment of topo I and topo II based on the torsional mechanics of chromatin. In addition, we show that cohesin loop boundaries coincide with regions of (+) torsion in the genome, indicating a role of torsion in helping cohesin to restructure the genome topology.

Accessibility is likely to greatly influence the readout of any torsion sensor that relies on DNA binding. Thus, establishing the zero-torsion baseline is an essential step and must be performed under a condition that keeps chromatin intact. While torsion sensors other than TMP have been applied to generate genomic maps, these sensors, such as biotin-functionalized psoralen derivatives^25–27^ and the bacterial protein GapR^52^, are larger in size than TMP, and thus, these sensors are likely also sensitive to DNA accessibility. Since the method we developed to obtain the zero-torsion state is not specific to any sensor, it may be applied to other torsion sensors. Additionally, although this study utilized budding yeast as a model organism, preparation of the zero-torsion state can be similarly applied to cells of other organisms. Our work shows a way to measure torsion more accurately and helps to lay a foundation for future quantitative studies of torsion in the genome.

## Supplementary Material

Supplement 1

## Data Availability

All data are available in the main text or the supplementary materials.
