# Supplementary material for: High-Resolution Genome-Wide Maps Reveal Widespread Presence of Torsional Insulation
Source: bioRxiv. 2025 Jan 4:2024.10.11.617876. Originally published 2024 Oct 13. Preprint. [Version 2] doi: 10.1101/2024.10.11.617876 (PMC11482950; doi:10.1101/2024.10.11.617876)
Supplement: 1 [file NIHPP2024.10.11.617876V2-supplement-1.pdf]

bioRxiv preprint doi: https://doi.org/10.1101/2024.10.11.617876; this version posted January 4, 2025. The copyright holder for this preprint (which was not certified by peer review) is the author/funder, who has granted bioRxiv a license to display the preprint in perpetuity. It is made available under aCC-BY 4.0 International license.

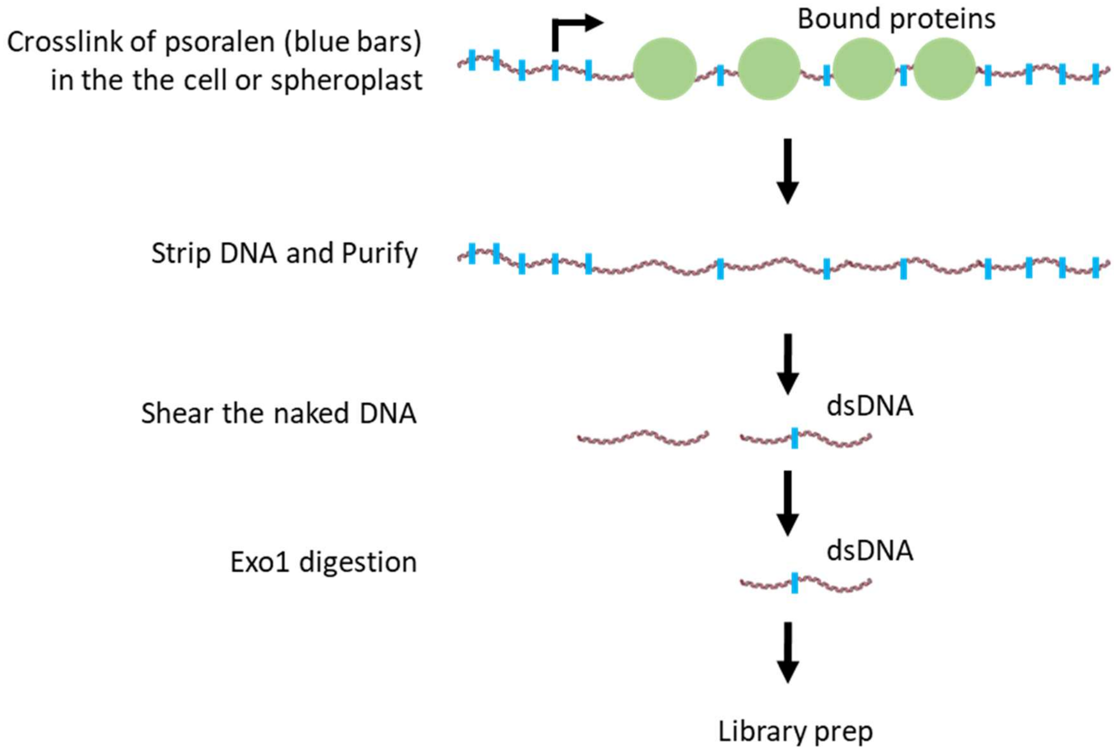

Figure Supplement 1. Psoralen binding and accessibility. Cartoon depicting the experimental method for mapping TMP cross-linking on genomic DNA. The TMP has preferential binding to naked DNA where there are no bound proteins, such as in nucleosome-free regions and on linker DNA.

bioRxiv preprint doi: https://doi.org/10.1101/2024.10.11.617876; this version posted January 4, 2025. The copyright holder for this preprint (which was not certified by peer review) is the author/funder, who has granted bioRxiv a license to display the preprint in perpetuity. It is made available under aCC-BY 4.0 International license.

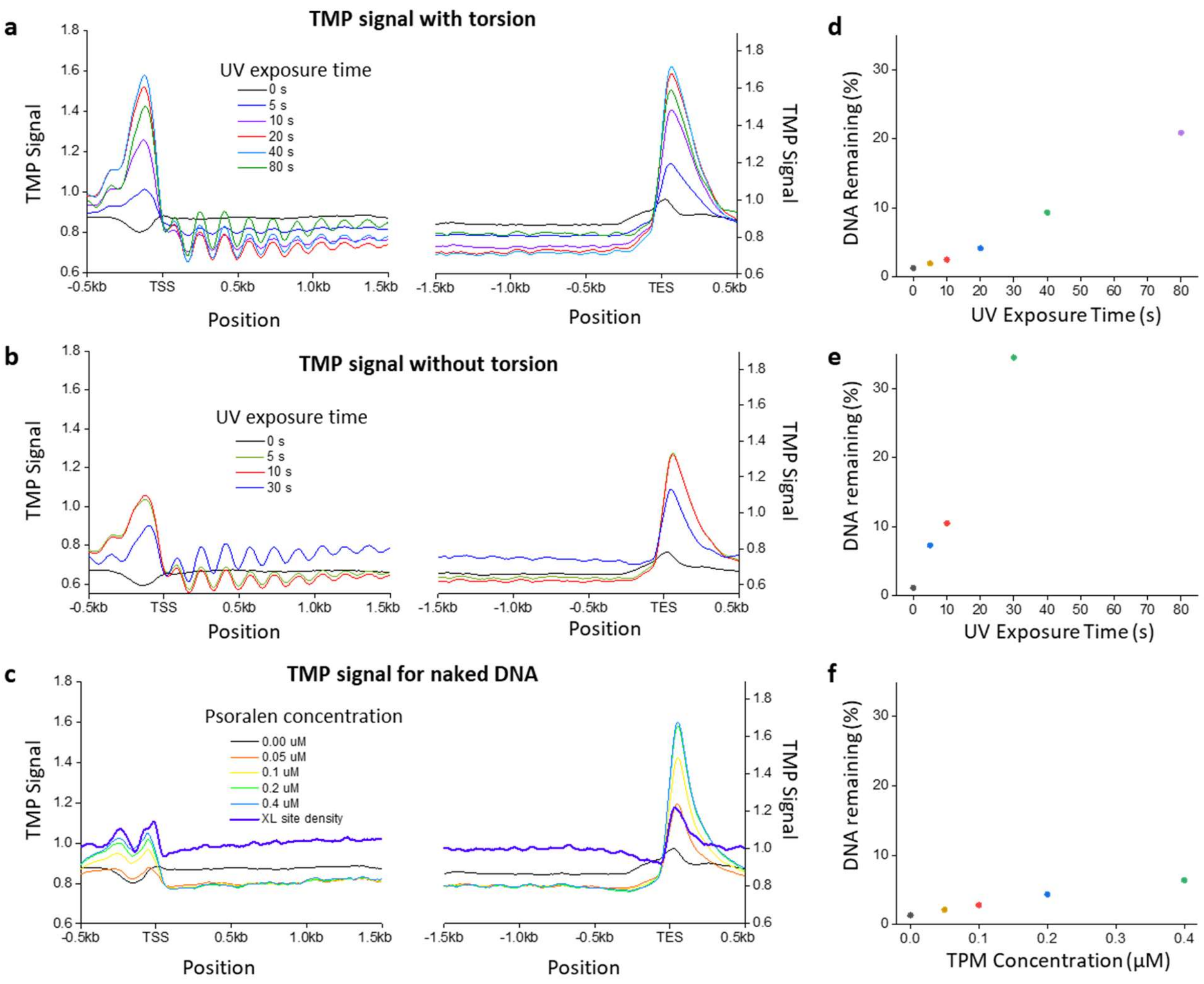

Figure Supplement 2. Psoralen Crosslinking Conditions.

(a) Average TMP signal with torsion of all 5925 genes as in Figure 1, shown for increasing UV dosage on cells. (Porter: did you divide by the purified DNA signal?)

(b) Average TMP signal without torsion from the same genes.

(c) Average TMP signal of naked genomic DNA from the same genes. 250 ng of sheared naked genomic DNA was incubated for 2 min with the indicated concentration of psoralen and exposed to UV light for 10 s. The density of adjacent AT or TA dinucleotide cross-linking sites is plotted for comparison with the naked DNA profiles (XL site density, dark blue).

(d-f) Percent of 250 ng of input DNA remaining after two rounds of denaturation and treatment with ExoI for each respective condition in (a-c). The response in % DNA remaining is near linear with UV dosage. At low concentrations, we see that the % DNA remaining approaches the background (d-f) as does the signal from the library prep (a-c). This indicates that if the dose is

bioRxiv preprint doi: https://doi.org/10.1101/2024.10.11.617876; this version posted January 4, 2025. The copyright holder for this preprint (which was not certified by peer review) is the author/funder, who has granted bioRxiv a license to display the preprint in perpetuity. It is made available under aCC-BY 4.0 International license.

too low, the signal-to-noise limit is being approached. Upon high UV exposure, the % DNA remaining indicates that there are likely multiple crosslinks/fragments (d,e), which may lead to artifacts due to the loss of small fragments during library prep. Upon a Goldilocks dose of UV exposure or psoralen concentration, signals are very similar to each other, indicating that most fragments have exactly one crosslink after Exo digestion (Fig. Supplement 3).

bioRxiv preprint doi: https://doi.org/10.1101/2024.10.11.617876; this version posted January 4, 2025. The copyright holder for this preprint (which was not certified by peer review) is the author/funder, who has granted bioRxiv a license to display the preprint in perpetuity. It is made available under aCC-BY 4.0 International license.

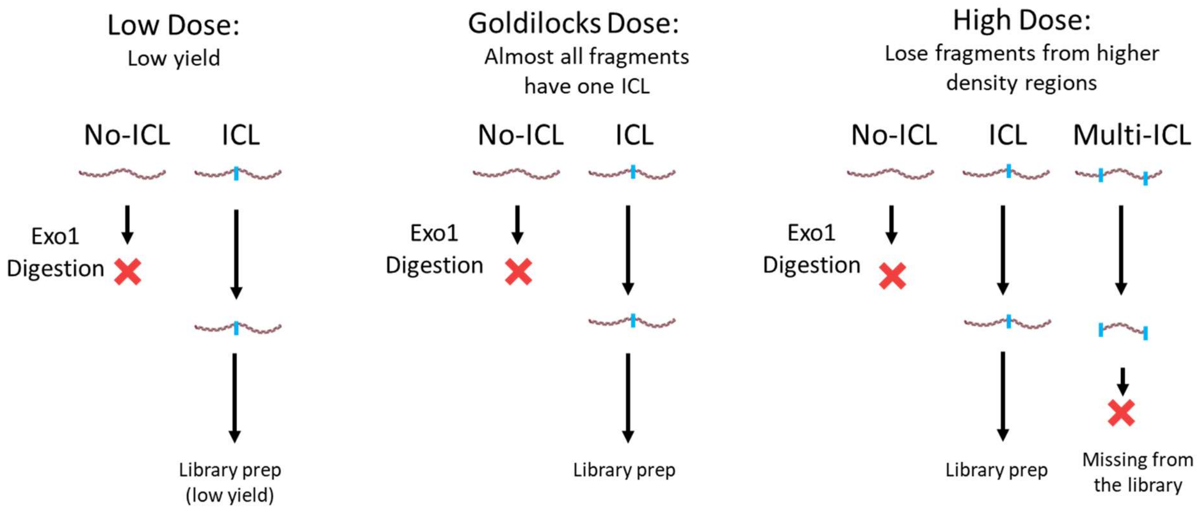

Figure Supplement 3. Optimal TMP crosslinking density. Cartoon depicting the optimal inter-strand cross-linking density is when each fragment of DNA has only one TMP cross-link after Exonuclease I digestion.
