# Supplementary material for: High-Resolution Genome-Wide Mapping of Chromatin Accessibility and Torsional Stress
Source: bioRxiv. 2024 Oct 13:2024.10.11.617876. Preprint. [Version 1] doi: 10.1101/2024.10.11.617876 (PMC11482950; doi:10.1101/2024.10.11.617876)
Supplement: Supplementary file 1 [file media-1.pdf]

Cell Culture

The *Saccharomyces cerevisiae* strain BY4741 (*MAT a his3Δ1 leu2Δ0 met15Δ0 ura3Δ0 bar1::NatMX* his3Δ1::HIS3-ADH1pr-hENT-GPDpr-HSV-TK) was used for all experiments. Cells were grown at 30°C in YPD media until the OD600 reached ~0.6, and then processed for different conditions as described below.

Zero torsion state preparation

Cells designated as zero torsion state controls were crosslinked with 1% (v/v) formaldehyde (Fisher) for 15 minutes at 30°C. Formaldehyde was quenched with 0.4 M glycine (Sigma) for 5 minutes at room temperature. Following quenching, cells were pelleted and washed 2X with PBS at 4˚C. Next, cells were spheroplasted via 0.01% (v/v) 10 mg/µl zymolyase (Zymo Research) treatment in 1 M sorbitol (Sigma), 0.5 mM 2-mercaptoethanol (Sigma) for 20 minutes. Spheroplasts were confirmed to be >90% of cells using microscopy. Formaldehyde-fixed spheroplasts were centrifuged at 1,000 g for 10 minutes at 4˚C, washed with 25 ml of cold PBS(Invitrogen), and resuspended in 100 µl of 1x DpnII reaction buffer (NEB) containing 100 units of DpnII (NEB) and supplemented with 0.1% (v/v) NP-40 (Sigma), and 10 µM TMP (Sigma). Samples were incubated for 2 hours at 37°C followed by TMP photobinding as described below.

Trimethylpsoralen Photobinding

The 4,5′, 8-trimethylpsoralen (TMP) (Sigma) was dissolved in 100% ethanol at a final stock solution of 2 mM. TMP was added to growing cells at a final concentration of 10 µM and cells were further incubated at 30°C for 1 hour. Twenty-five mL of cell solution was placed in a 10 cm petri dish, and exposed to ~0.15 W/cm^2 of 365-nm light for 20 seconds. Directly after TMP photobinding, the cells were fixed in 95 ml 70% -20 °C ethanol and placed at -20°C overnight. Cells were then harvested by centrifugation at 10,000 g for 10 minutes, resuspended in 500 µl of 50 mM Sodium citrate (Sigma), pH 7.4, and incubated at room temp for 10 minutes. Centrifugation and wash steps were repeated once more, followed by a 20-minute zymolyase treatment as detailed above. Spheroplasts were harvested by centrifugation and resuspended in 100 µl of lysis buffer (300 mM NaCl (Sigma), 50 mM EDTA (Sigma), 2% SDS (Sigma), and 5 % (v/v) Rnase A(Fisher) for ~2 hours and then vortexed for 2 minutes. 10 µl of Proteinase K (NEB) was added, and cells were incubated for 2 hours at 65 °C.

Zero torsion samples followed a similar photobinding protocol but were exposed to UV light at ~0.35 W/cm^2 for 10 seconds in a 1.5 ml Eppendorf tube. Samples were harvested at 10,000 g at 4°C, the supernatant was removed, and they were resuspended in 100 µl of Lysis buffer containing 10 µl Proteinase K. The zero torsion samples were incubated at 65°C overnight to reverse formaldehyde crosslinks. After lysis, genomic DNA was extracted from all samples via 2X phenol-chloroform extraction followed by isopropanol precipitation.

TMP-seq

The TMP-seq protocol was adapted from previous work[1,2]. Extracted genomic DNA was sonicated to ~400 bp fragments using appropriate settings on a Covaris E200 sonicator. Following sonication, DNA was treated with 5 ul RNase A (Invitrogen) and purified using a Zymoclean DNA clean and concentrator -5 kit according to the manufacturer's protocol using a 7:1 binding buffer:sample ratio. After quantification using a Biotum HS DNA quantification kit, ~250 ng of DNA was diluted into 86 µl of 10 mM Tris pH 8.5, incubated at 98°C for 15 minutes, and then directly moved to ice for 2 minutes. DNA was then digested with 4 µl of ExoI (NEB) in 1X ExoI reaction buffer at 37°C for 1 hour. This digestion process was repeated by incubating at 98°C for 1 hour and then adding an additional 4 µl of ExoI followed by another incubation at 37°C for 1 hour. Digested samples were purified using a Zymo DNA clean and concentrator -5 kit using a 7:1 binding buffer: sample ratio. DNA was again quantified using the Biotum HS DNA assay. Illumina adaptors were ligated to the digested samples using the NEBNext Ultra II DNA library prep kit for Illumina (NEB) at half the standard total reaction volume of the manufacturer's protocol. Adapter ligated DNA was purified with 2X Ampure beads (Beckman Coulter) and eluted in 40 µl of 10 mM Tris buffer pH 8.5. DNA was stored at -20 C until further processing.

To locate Psoralen inter-strand crosslinks, DNA from the previous step was incubated with 5 µl of lambda exonuclease (NEB) in the provided buffer in a 50 ul total reaction volume at 37°C for 1 hour, purified with 1.8X Ampure beads, and eluted with 30 µl of 10 mM Tris pH 8.5. Lambda-digested DNA was then subjected to 10 rounds of primer extension with Q5 DNA polymerase (NEB), using 10 nM of the primer: 5'-GTGACTGGAGTTCAGACGTGTGCTCTTCCGATC*T on a thermocycler (98°C for 10s → 10 rounds (98°C, 10s → 65°C, 2 min)→ 65°C, 5min → 95°C 1min → 4°C). Products were purified using 1.8x Ampure beads and eluted in 40 µl 10mM Tris pH 8.5. Primer extension products were ribotailed using 1.4 µl Terminal Transferase (NEB) and 0.4 mM rGTP (Roche) in Terminal Transferase buffer supplemented with 0.25 mM $CoCl_2$ by incubating at 37°C for 30 minutes followed by incubation at 75°C for 20 min. Ribotailed products were purified with 1.8X Ampure beads, eluted with 30 µl 10mM Tris pH 8.5. Purified ribotailed products were ligated with 0.33 µM annealed adapter oligos (Top: 5'p-AGA TCG GAA GAG CGT CGT GTA GGG AAA GAG TGT-3', Bottom: 5' ACA CTC TTT CCC TAC ACG ACG CTC TTC CGA TCT CCC -3') using 1.2 ul T4 DNA ligase (NEB) in the provided buffer for 1 hour at 16 °C, and subsequently purified with 1.8X Ampure beads and eluted in 20 µl 10mM Tris pH 8.5. Finally, products were indexed and amplified using NEBNEXT Multiplex Oligos for Illumina set 1 following the manufacturers protocol with a total of 12 PCR cycles. The PCR products were purified using 0.8X Ampure beads and eluted in TE (Invitrogen). Samples were pooled at equimolar concentrations and empty adapters were removed from the pool.

Sequencing and Data Processing

Pooled libraries were sequenced on a NextSeq 2000 (Illumina) using a NextSeq 2K P2 100 bp kit (Illumina) in paired-end mode with a 63 bp Read_1 and 59 bp Read_2. Sequencing, demultiplexing and Fastq generation was performed by the Cornell Biotechnology Resource Center. Fastq files were aligned to the SacCer3 Genome using BWA-mem2[3] with default settings for paired-end reads. Sam files were converted to BAM files followed by fixmate and duplicate marking Samtools[4]. Duplicates were again marked using picard-MarkDuplicates[5], and duplicates were removed using Samtools-view. Bedgraph files were generated using deeptools[6] bamCoverage with 10 bp bins using the following settings (--samFlagInclude 64 --Offset 1) yielding the coverage of the 5' end of Read_1 where the ICL was located.

Bedgraph files were further processed in MATLAB to ensure one bin per 10 bp interval, and normalized to the mean number of reads per bin. Replicate bedgraphs were averaged, and indicated subtractions were done using MATLAB. Normalized bedgraphs were converted to bigwig files using ucsc-bedGraphToBigWig[7]. Heatmaps and composite plots were generated using deeptools computeMatrix, and visualized using Origin Lab. Bed files containing coordinates of verified *s. cerevisiae* ORFs were downloaded from the Saccharomyces Genome Database (SGD)[8,9], and a region blacklist was downloaded from the yeastepigenome.org[10]. Bed files for each gene configuration in Figure 3 were generated by considering the boundaries of nearest neighbor verified ORFs and classifying them based on their strand configuration. Regions that had any annotations according to the list of all yeast genes from the SGD were removed to isolate true intergenic regions. The resulting Bed files were used to make heatmaps and composite plots as described above.

External Data Processing

BAM files of ChIP-Exo, and H3-ChIP-MNase data was downloaded from yeastepigenome.org[10]. For ChIP exo, bigwig files were calculated using the 5' end of read 1 as in[10]. Signals were then normalized to the mean number of reads per 10bp bin. For H3-ChIP-MNase , the center of the fragment was considered. Top1 and Top2 ChIP-Exo data were further processed by subtracting the No-TAP control (ChIP-Exo background). Previous Scc1 ChIP-seq IP and Input DNA data was processed by downloading bigwig files from GSE235560[11]. Deeptools bigwigCompare was used to normalize Scc1 ChIP/Input DNA.

mRNA-seq data was downloaded from GSE76142[12] in bigwig format. The minus strand signal was subtracted from the plus strand signal. Expression level for a gene was calculated using mean absolute value of the subtracted signal within the ORF, bed files containing expression levels were used to separate data into percentile groups.

To determine positions of intrachromosomal loop boundaries and their scores, mCool files of MicroC-XL data were downloaded from GSE151553[13]. Boundaries were located and scored using cooltools dots[14] with a 500 bp bin size. Loop calls were refined by only considering the smallest loops in nested configurations, thereby only representing each region once. Boundary

positions were adjusted by locating the position of the nearest (<500 bp) Scc1-ChIP-seq peak on either loop boundary.

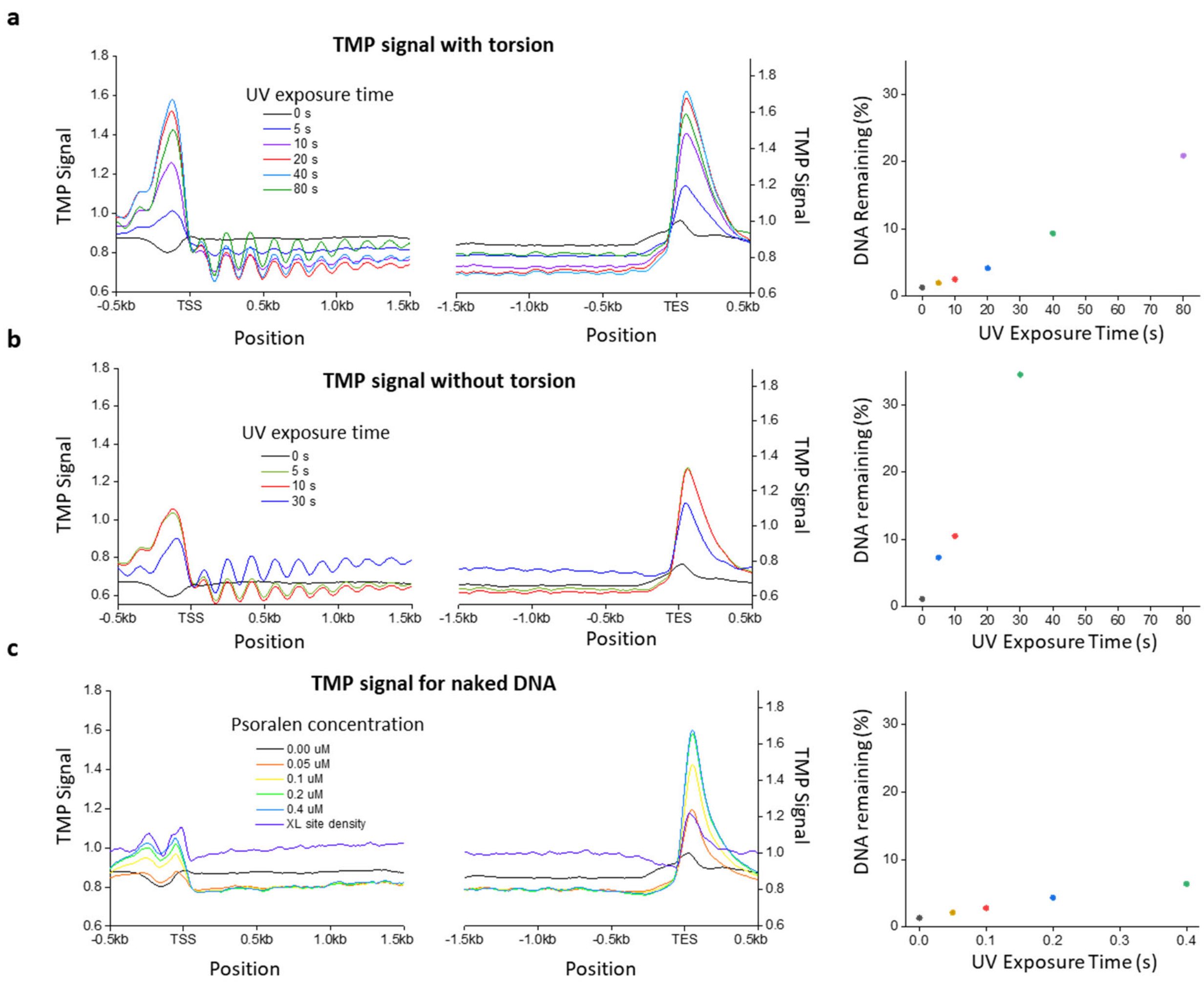

Figure S1. Psoralen Crosslinking Conditions.

(a) Average TMP signal with torsion of all 5925 genes as in Figure 1, shown for increasing UV dosage on cells.

(b) Average TMP signal without torsion from the same genes.

(c) Average TMP signal of naked genomic DNA from the same genes. 250 ng of sheared naked genomic DNA was incubated for 2 min with the indicated concentration of psoralen and

exposed for to UV light for 10 s. The density of adjacent AT or TA dinucleotide cross-linking sites is plotted for comparison with the naked DNA profiles (XL site density, dark blue).

(d-f) Percent of 250 ng of input DNA remaining after two rounds of denaturation and treatment with ExoI for each respective condition in (a-c). The response in % DNA remaining is near linear with UV dosage or TMP concentration. At low concentrations or dosages, we see that the % DNA remaining approaches the background (d-f) as does the signal from the library prep (a-c). This indicates that if the dose is too low, the signal-to-noise limit is being approached. Upon high UV exposure, the % DNA remaining indicates that there are likely multiple crosslinks/fragments (d,e), which may lead to artifacts due to the loss of small fragments during library prep. Upon a Goldilocks dose of UV exposure or psoralen concentration, signals are very similar to each other, indicating that most fragments have exactly one crosslink after Exo digestion (Fig. S3).

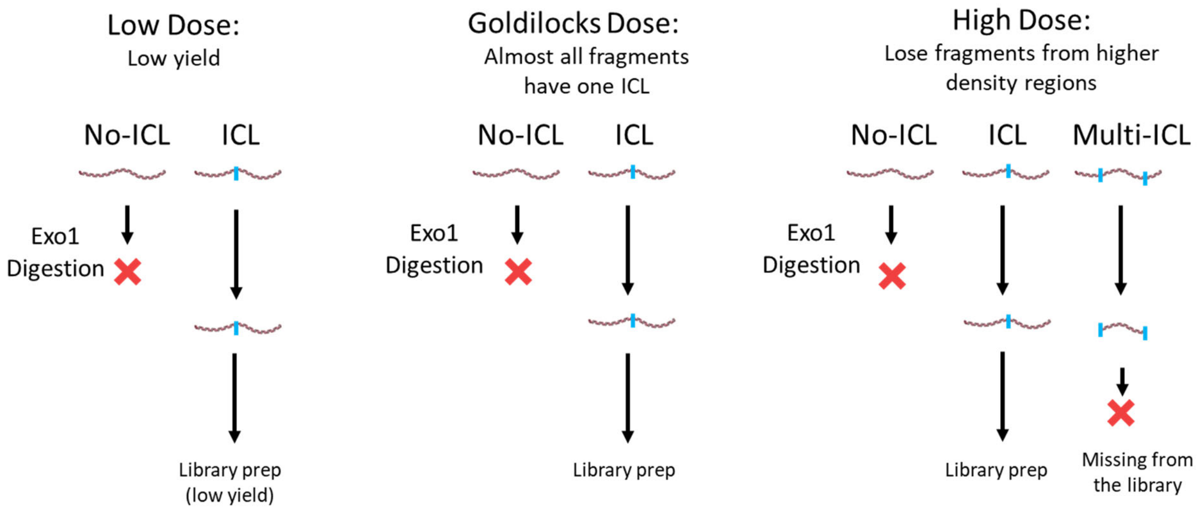

Figure S2. Optimal TMP crosslinking density. Cartoon depicting the optimal inter-strand cross-linking density is when each fragment of DNA has only one TMP cross-link after Exonuclease I digestion.

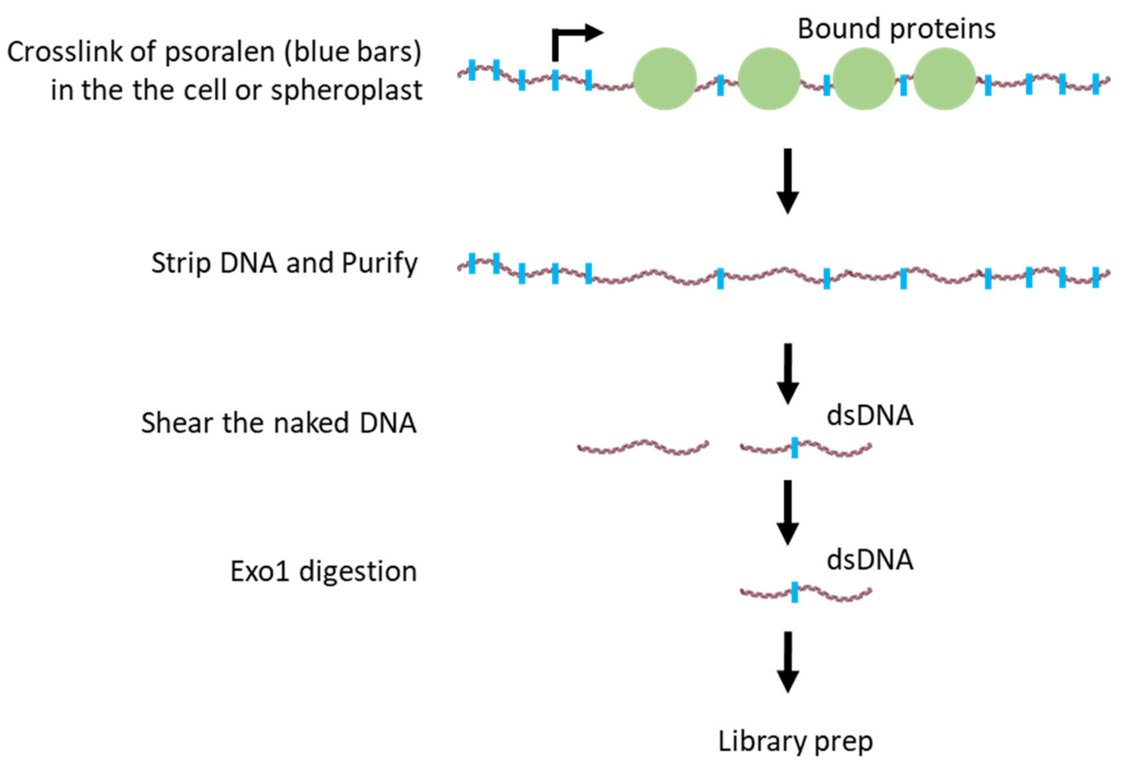

Figure S3. Psoralen binding and accessibility. Cartoon depicting the experimental method for mapping TMP cross-linking on genomic DNA. The TMP has preferential binding to naked DNA where there are no bound proteins, such as in nucleosome-free regions and on linker DNA.

References

1 Teves, S. S. & Henikoff, S. Transcription-generated torsional stress destabilizes nucleosomes. *Nature structural & molecular biology* **21**, 88-94 (2014).

2 Bermúdez, I., García-Martínez, J., Pérez-Ortín, J. E. & Roca, J. A method for genome-wide analysis of DNA helical tension by means of psoralen-DNA photobinding. *Nucleic Acids Res* (2010).

3 Vasimuddin, M., Misra, S., Li, H. & Aluru, S. in *2019 IEEE international parallel and distributed processing symposium (IPDPS).* 314-324 (IEEE).

4 Danecek, P. *et al.* Twelve years of SAMtools and BCFtools. *Gigascience* **10**, giab008 (2021).

5 *Picard Tools*, <https://broadinstitute.github.io/picard/> (

6 Ramírez, F. *et al.* deepTools2: a next generation web server for deep-sequencing data analysis. *Nucleic Acids Research* **44**, W160-W165 (2016).

7 Raney, B. J. *et al.* The UCSC genome browser database: 2024 update. *Nucleic Acids Research* **52**, D1082-D1088 (2024).

8 Cherry, J. M. *et al.* Saccharomyces Genome Database: the genomics resource of budding yeast. *Nucleic acids research* **40**, D700-D705 (2012).

9 Sayers, E. W. *et al.* Database resources of the national center for biotechnology information. *Nucleic acids research* **50**, D20-D26 (2022).

10 Rossi, M. J. *et al.* A high-resolution protein architecture of the budding yeast genome. . *Nature* **592**, 309-314 (2021).

11 Jeppsson, K. *et al.* Loop-extruding Smc5/6 organizes transcription-induced positive DNA supercoils. *Molecular Cell* **84**, 867-882. e865 (2024).

12 Booth, G. T., Wang, I. X., Cheung, V. G. & Lis, J. T. Divergence of a conserved elongation factor and transcription regulation in budding and fission yeast. *Genome research* **26**, 799-811 (2016).

13 Costantino, L., Hsieh, T. H. S., Lamothe, R., Darzacq, X. & Koshland, D. Cohesin residency determines chromatin loop patterns. *eLife* **9**, 1-31 (2020).

14 Open2C *et al.* Cooltools: enabling high-resolution Hi-C analysis in Python. *PLOS Computational Biology* **20**, e1012067 (2024).
